# An infant formula containing dairy lipids increased red blood cell membrane Omega 3 fatty acids in 4 month-old healthy newborns: a randomized controlled trial

**DOI:** 10.1186/s12887-018-1047-5

**Published:** 2018-02-13

**Authors:** Maria Lorella Gianni, Paola Roggero, Charlotte Baudry, Catherine Fressange-Mazda, Claudio Galli, Carlo Agostoni, Pascale le Ruyet, Fabio Mosca

**Affiliations:** 1 Neonatal Intensive Care Unit (NICU), Department of Clinical Science and Community Health, Fondazione IRCCS “Ca’ Granda” Ospedale Maggiore Policlinico, University of Milan, Milan, Italy; 2 Nutrition Department, Lactalis R&D, Retiers, France; 3 Lactalis Nutrition Europe, Torcé, France; 4 University of Milan, Milan, Italy; 5 Pediatric Intermediate Care Unit, Department of Clinical Science and Community Health, Fondazione IRCCS “Ca’ Granda” Ospedale Maggiore Policlinico, University of Milan, Milan, Italy

**Keywords:** Infant nutrition, Infant formula, Lipid quality, Dairy fat, Fatty acids, Omega 3, Red blood cells membrane fatty acids, Erythrocyte membrane fatty acids

## Abstract

**Background:**

When breastfeeding is not possible, infants are fed formulas (IF) in which lipids are usually of plant origin. However, the use of dairy fat in combination with plant oils enables a lipid profile closer to breast milk in terms of fatty acid (FA) composition, triglyceride structure, polar lipids and cholesterol contents. The objective of this study was to determine the effect of an IF containing a mix of dairy fat and plant oils on Omega-3 FA content in red blood cells (RBC).

**Methods:**

This study was a monocentric, double-blind, controlled, randomized trial. Healthy term infants were fed formulas containing a mix of dairy fat and plant oils (D), plant oils (P) or plant oils supplemented with ARA and DHA (PDHA). Breastfed infants were enrolled as a reference group (BF). FA in RBC phosphatidylethanolamine was evaluated after 4 months and FA in whole blood were evaluated at enrollment and after 4 months by gas chromatography. Differences between groups were assessed using an analysis of covariance with sex and gestational age as covariates.

**Results:**

Seventy IF-fed and nineteen BF infants completed the protocol. At 4 months, RBC total Omega-3 FA levels in infants fed formula D were significantly higher than in group P and similar to those in groups PDHA and BF. RBC DHA levels in group D were also higher than in group P but lower than in groups PDHA and BF. RBC n-3 DPA levels in group D were higher than in groups P, PDHA and BF. A decrease in proportions of Omega-3 FA in whole blood was observed in all groups.

**Conclusions:**

A formula containing a mix of dairy lipids and plant oils increased the endogenous conversion of Omega-3 long-chain FA from precursor, leading to higher total Omega-3, DPA and DHA status in RBC than a plant oil-based formula. Modifying lipid quality in IF by adding dairy lipids should be considered as an interesting method to improve Omega-3 FA status.

**Trial registration:**

Identifier NCT01611649, retrospectively registered on May 25, 2012.

## Background

Breast milk is considered by the World Health Organization (WHO) as the optimal form of nourishment for infants up to 6 months. Lipids are major components in human milk, providing approximately 50% of total dietary energy. Triacylglycerols in human milk have a specific fatty acid (FA) composition, which can be influenced by maternal dietary habits or by lactation stages. Proportions of saturated and monounsaturated FA are relatively stable whereas proportions of polyunsaturated FA (PUFA) can vary more widely [1]. Among PUFA, human milk provides alpha-linolenic (ALA) and linoleic acids (LA) which are essential fatty acids (EFA). They can be endogenously converted by the newborn into long-chain derivatives of Omega 3 and Omega 6 families, such as docosahexaenoic acid (DHA) and arachidonic acid (ARA), respectively. However, this conversion is considered to be low in humans [2]. Human milk also contains preformed long-chain PUFA (LCPUFA) of both series with DHA representing 0.1–1% of total FA and ARA 0.4–0.9% of total FA [1]. LCPUFA accumulation is very important during both the fetal and postnatal periods in brain and retina [3]. Consequently, bioavailability of ALA, LA, DHA and ARA from breast milk or infant formulas is critical in early life to sustain optimal visual and brain development of the newborn and later-life cognitive functions [4].

Fat sources used in most infant formulas currently marketed are blends of plant oils used to match the FA profile found in human milk. Indeed, since the middle of 20th century, infant formulas have been enriched in EFA-rich plant oils and bovine milk fat has been progressively removed [1]. Infant formulas can also be optionally supplemented with fish, algae or fungus oils providing preformed DHA and ARA. However, lipids of plant oils are not comparable to lipids of human milk in terms of FA diversity, triacylglycerol structure, fat globule composition, complex lipids and cholesterol contents. Although not containing high levels of LCPUFA, the use of dairy lipids in combination with plant oils could provide a lipid composition and structure closer to human milk, thus improving the quality of infant formula fat profile. Indeed, infant formulas provide milk-specific FA and cholesterol only when dairy lipids are used as a fat source in combination with plant oils [1]. Short and medium chain FA, lauric acid, myristic acid and palmitic acid contents in formulas can vary considerably according to the use of palm oil, coconut oil or dairy lipids [1]. Moreover, plant oils do not encounter the specific triglyceride structure with palmitic acid in sn2 position found in breast milk (60–85% of sn2-palmitate) or cow’s milk (more than 45%). Therefore, it has been shown that formulas with only plant oils as sources of lipids had less than 15% of palmitic acid in sn2 position whereas formulas containing a blend of dairy lipids and plant oils had 48% of palmitic acid in sn2 position [5]. This triglyceride structure is of particular importance because long-chain saturated FA in the sn2 position are more efficiently digested and absorbed [6].

Dairy lipids specifically contain about 10% of myristic acid and 10% of short and medium chain FA (C4-C10) while plant oils provide much less of these FA. Short-chain FA represent a rapid source of energy for the infant, because they enter directly the portal vein and are known to be completely absorbed and metabolized. Myristic and short/medium chain FA could also increase bioavailability and conversion of n-3 PUFA as demonstrated in vitro [7]. Furthermore, benefits of dairy lipids on the conversion of Omega 3 precursor to long-chain derivatives have been described in several studies. Indeed, in adults, a moderate intake of dairy lipids and rapeseed oil improved plasma DHA levels and blood fluidity [8, 9]. In Omega 3-deficient rats, it has been demonstrated that for a similar ALA content, a blend of dairy lipids and rapeseed oil induced a higher level of brain DHA than a blend of plant oils even if supplemented with preformed DHA [10, 11]. In mice, a diet containing a blend with dairy lipids given from the first day of gestation until adulthood increased DHA levels and neuroplasticity in the brain of the offspring compared with a diet containing only plant oils [12]. In the same mouse model, it has been also demonstrated that a diet containing dairy lipids protected from the adverse effects induced by an early life inflammatory event on neurogenesis and adult spatial memory [13]. Consequently, these data suggest that the use of dairy lipids in combination with plant oils could potentially improve long-chain Omega 3 status and promote the accretion of DHA in the developing brain.

The aim of the present study was to assess the impact of an infant formula containing dairy lipids and plant oils on FA composition in RBC and whole blood, especially on Omega 3 FA content, in 4 month-old healthy infants.

## Methods

### Study design

This monocentric, double-blind, controlled, randomized trial was conducted in 2012–2013 in the Department of Neonatology of the Fondazione IRCCS Cà Granda Ospedale Maggiore Policlinico, Milano, Italy (NCT01611649). The study was approved by the local Ethical Committee and conducted in accordance with Good Clinical Practice and the principles and rules of the Declaration of Helsinki. Parents or legal caregivers provided written informed consent prior to enrollment of their infants in the study. The study protocol has been previously published [14].

### Population

Healthy full-term infants born in the Department of Neonatology were screened for participation in the study. When breastfeeding was not possible (contraindication or mothers not intended to breastfeed), infants were randomly allocated to be fed for 4 months with a formula containing either: a mix of plant oils and dairy fat (D), only plant oils (P) or plant oils supplemented with ARA and DHA (PDHA). Infants whose mothers intended to exclusively breastfeed from birth through at least 4 months were enrolled in a nonrandomized reference group (BF). Inclusion criteria were: gestational age 37 to 42 weeks, birth weight > 2500 g, healthy newborns from normal pregnancy, aged up to 3 weeks when entering the study, no breastfeeding (for the formula-fed groups) or exclusive breastfeeding (for the reference group). Exclusion criteria were: positive family history of allergy to milk proteins, known congenital or postnatal diseases which could interfere with the study and newborns whose parents had planned to move within 6 months after birth.

### Study formulas

Study formulas were formulated into powders and were reconstituted at 13.3%. They were manufactured and provided by Milumel®, Lactalis, Craon, France. The 3 tested formulas were packaged in blinded containers labeled only with study details and number of randomization; they were indistinguishable in appearance and texture. Both the investigators and the infants’ parents were blind to the group allocation. The randomization schedule was computer-generated and stratified on sex. Compositions of the 3 study formulas were in compliance with the European Directive 2006/141/EC on infant formulas and are detailed in Table 1. The 3 formulas had similar energy and macronutrient contents but they differed by the nature of their lipid sources. Formula D contained a mixture of dairy lipids and plant oils (rapeseed, sunflower, high oleic sunflower); formula P contained only plant oils (palm, rapeseed, sunflower) and formula PDHA contained plant oils (palm, rapeseed, sunflower) supplemented with ARA (0.4%) and DHA (0.2%). Study formulas were consumed straightaway after randomization and were provided for the 4 subsequent months.

**Table 1:** Composition of study formulas

		Formula D	Formula P	Formula PDHA
		100 ml^a^	100 ml^a^	100 ml^a^
Energy	kJ	275	275	275
	kcal	66	66	66
Protein	g	1.3	1.3	1.3
Carbohydrates	g	8.1	8.1	8.1
Lactose	g	6.8	6.8	6.8
Fat	g	3.1	3.1	3.1
LA	mg	439	549	549
	% TFA	14.2	17.7	17.7
ALA	mg	73	55	55
	% TFA	2.3	1.8	1.8
Ratio LA/ALA		6	10	10
ARA	mg	≤ 0.1		12.4
	% TFA			0.4
DHA	mg			6.2
	% TFA			0.2
Ratio ARA/DHA				2

Composition of tested formulas according to manufacturer data. Formula D contained a mixture of dairy lipids (cream) and plant oils (rapeseed, sunflower and high oleic sunflower); formula P contained only plant oils (palm, rapeseed, sunflower) and formula PDHA contained plant oils (palm, rapeseed, sunflower) supplemented with ARA and DHA)

*LA* linoleic acid, *ALA* alpha-linolenic acid, *ARA* arachidonic acid, *DHA* docosahexaenoic acid, *TFA* total fatty acids

^a^Reconstituted 13.3%

### Objectives and outcomes

The main objective of this study was to investigate the effect of formula D on the RBC membrane total Omega 3 FA content as compared to formulas P and PDHA and to BF reference group. Total Omega 3 FA levels included ALA, EPA (eicosapentaenoic acid, 20:5n-3), DPA (docosapentaenoic acid, 22:5n-3) and DHA levels. The secondary objective was to evaluate changes between 4 months and enrollment in whole blood FA contents in infants consuming formula D in comparison to formula P and PDHA and BF. Impact of formulas on growth, body composition and gastrointestinal tolerance was also investigated but these data are under publication in another article.

### Measurement of FA levels in RBC membrane

Venous blood samples were drawn after consumption of the allocated formula or breastfeeding for 4 months. Blood was collected on heparin. Plasma was separated by 15-min centrifugation (2200 g at 4 °C) from RBC that were rinsed with saline solution (NaCl 0.9%). Plasma and RBC were stored at − 80 °C for later analysis. RBC total lipids were extracted and phosphatidylethanolamine (PE) was isolated from other phospholipids by thin layer chromatography. FA levels of RBC PE were measured by means of a gas–liquid chromatography (HPLC) by ITERG, Pessac, France. Results are expressed as percentage of total FA.

### Measurement of blood FA levels

Whole blood samples were collected using a heel stick at enrollment and after 4 months of consumption of the allocated formula or breastfeeding. FA levels were quantified by means of a gas–liquid chromatography (HPLC) by Oxigenlab, Brescia, Italy. Results are expressed as percentage of total FA.

### Sample size

Calculation of sample size for this study has been previously detailed [14]. In order to detect a difference of 20% in the RBC membrane Omega 3 FA levels (basal value of 7.3% and standard deviation of 1.3 [15]) between infants receiving formula D and infants receiving formula P or formula PDHA, at a power of 90% and with an alpha error of 5%, a total of 17 infants per group was necessary. To take into account the multiplicity of comparisons, the calculation was done with an alpha error of 2.5% and a total of 21 infants per group was needed. Considering dropouts, a total of 30 infants per group were enrolled.

### Statistical analyses

Statistical analyses were performed using SAS software (SAS Institute Inc., Cary NC, USA) by Soladis, Lyon, France. Continuous variables were expressed as mean and standard deviation. Differences among groups for FA levels were analyzed with an analysis of variance with 3 fixed factors (group, sex, gestational age). Pairwise comparisons were made using post-hoc Tukey’s test for multiple comparisons. A *p*-value < 0.05 was considered significant.

## Results

### Study population

**Fig. 1:**
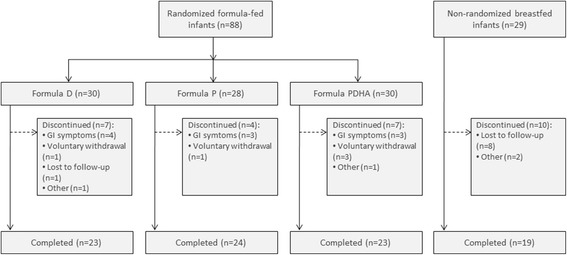
Flow of study subjects. Formula D contained a mixture of dairy lipids and plant oils; formula P contained only plant oils and formula PDHA contained plant oils supplemented with ARA and DHA. GI (gastrointestinal) symptoms included regurgitation, reflux, constipation, colic and flatulence

A total of 117 healthy newborns were enrolled. Of these, 88 were randomized to the formula-fed groups and 29 were enrolled in the breastfed reference group (Fig. 1). A total of 18 (20%) infants from the formula groups and 10 (34%) in the breastfed group withdrew before the end of the study (Fig. 1). Rates of discontinuation were similar in the 3 formula groups (23% in formula D and PDHA; 14% in formula P). Gastrointestinal symptoms (ie. regurgitations, reflux, constipation, colic and flatulence) were the most common reason for study discontinuation in the 3 formula groups: 57% in group D, 75% in group P and 43% in group PDHA. Among the breastfed group, 8 babies were lost to follow-up and breastfeeding was stopped before the next visit for 2 babies.

Baseline characteristics in each group are presented in Table 2. Proportion of boys was similar in the 3 formula groups (53–57%) and was of 38% in the breastfed reference group (Table 2). A high proportion of caesarean births (43–64%) was observed in all groups without any difference between groups (Table 2). Gestational age was around 38 weeks in the 3 formula groups and around 39 weeks in the breastfed group. On average, infants were enrolled in the study at 5–10 days of life (Table 2). At baseline, weight in group P was significantly lower than in breastfed infants (*p* = 0.015) (Table 2). Height, head circumference and body composition were similar in the 4 groups at baseline (all *p* > 0.05) (Table 2). Total Omega 3 and DHA levels in whole blood were similar in the 4 groups at baseline, despite a tendency for higher DHA levels in the BF group but not statistically significant (Table 2).

**Table 2:** Infants’ characteristics at enrollment

	Formula D	Formula P	Formula PDHA	Breastfed	*p*-value
	*n* = 30	*n* = 28	*n* = 30	*n* = 29	
Sex, no. (%)					
Girls	13 (43.3)	11 (39.3)	14 (46.7)	18 (62.1)	0.329
Boys	17 (56.7)	17 (60.7)	16 (53.3)	11 (37.9)	
Delivery mode, no. (%)					
Natural	11 (36.7)	10 (35.7)	17 (56.7)	14 (48.3)	0.310
Caesarean	19 (63.3)	18 (64.3)	13 (43.3)	15 (51.7)	
Gestational age, weeks, mean (SD)	38.4 (1.2)	38.4 (1.1)	38.3 (1.0)	39.0 (1.0)	0.072
Age at enrollment, days, mean (SD)	6.8 (5.6)	4.9 (4.5)	9.6 (7.9)	8.8 (6.6)	0.076
Weight, g, mean (SD)	3144.2 (448.6)	2942.9 (386.6)^X^	3156.4 (431.8)	3308.7 (533.9)	0.029
Height, cm, mean (SD)	49.1 (2.2)	48.9 (2.2)	49.7 (1.9)	50.1 (1.8)	0.112
Head circumference, cm, mean (SD)	34.4 (1.2)	34.3 (1.4)	34.7 (1.4)	35.3 (2.0)	0.068
Fat mass, % BW, mean (SD)	10.3 (2.9)	10.8 (3.4)	9.8 (3.5)	11.6 (5.4)	0.321
Total Omega 3 in whole blood, %, mean (SD)	12.9 (3.1)	14.2 (3.6)	13.2 (4.2)	15.0 (4.3)	0.384
DHA in whole blood, %, mean (SD)	3.2 (1.1)	3.6 (1.1)	3.4 (0.9)	4.1 (1.8)	0.057

Formula D contained a mixture of dairy lipids and plant oils; formula P contained only plant oils and formula PDHA contained plant oils supplemented with ARA and DHA. BW: body weight. Comparison of the 4 groups by ANOVA 1 fixed factor (Group) and post-hoc Tukey’s adjustments

^X^*p* < 0.05 vs. BF

Mean daily volume of formula consumed was evaluated during a 2 day-period at 1 month and 3 months. Most of formula-fed infants consumed more than 600 ml daily at 1 month (around 95% of infants) and more than 700 ml daily at 3 months (around 82%). No significant differences in formula intake were observed among the 3 formula groups at 1 month (*p* = 0.980) or at 3 months (*p* = 0.177).

### RBC membrane FA levels after 4 months

**Fig. 2:**
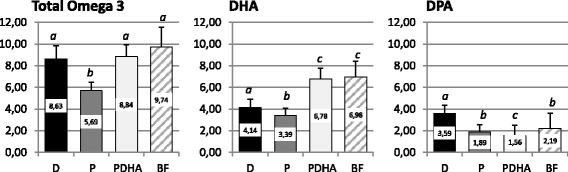
Total Omega 3, DHA and DPA levels (% of total FA) in PE of RBC membrane after 4 months. Formula D (*n* = 23) contained a mixture of dairy lipids and plant oils; formula P (*n* = 24) contained only plant oils and formula PDHA (*n* = 23) contained plant oils supplemented with ARA and DHA. BF: breastfed reference group (*n* = 18). PE: phosphatidylethanolamine. Comparison of the 4 groups by ANOVA with 3 fixed factors (Sex, gestational age, group) and post-hoc Tukey’s adjustments. *a ≠ b ≠ c; p < 0.05*

Primary endpoint was total Omega 3 FA levels in PE of RBC membrane after 4 months (Fig. 2). RBC total Omega 3 FA levels were significantly higher in infants fed with formula D (8.6 ± 1.2% TFA) than with formula P (5.7 ± 0.8% TFA; *p* <  0.001) but similar to those of infants fed with formula PDHA (8.8 ± 1.1% TFA; *p* = 0.956) or BF (9.7 ± 1.8% TFA; *p* = 0.191) (Fig. 2). Sum of DHA and n-3 DPA levels accounts for more than 90% of RBC total Omega 3. DHA levels in group D (4.1 ± 0.8% TFA) were significantly higher than in group P (3.4 ± 0.7% TFA; *p* = 0.029) but lower than in groups PDHA (6.8 ± 1.0% TFA) and BF (7.0 ± 1.4% TFA; both *p* <  0.001) (Fig. 2). n-3 DPA levels in group D (3.6 ± 0.6% TFA) were higher than in all the other groups (P: 1.9 ± 0.3; PDHA: 1.6 ± 0.2; BF: 2.2 ± 0.5% TFA; *p* <  0.001 for all) (Fig. 2). In group PDHA, total Omega 3 and DHA levels were similar to breastfed but n-3 DPA levels were significantly lower (*p* <  0.001).

When the BF group was excluded from the statistical comparison, results were similar with significantly higher levels of total Omega 3 and DHA in group D than in group P (*p* <  0.001 and *p* = 0.006, respectively).

RBC membrane compositions for the other FA are presented in Table 3. Total saturated FA were similar in the 3 formula groups. Percentage of MUFA was significantly higher in formula D-fed infants than in formula PDHA (*p* <  0.001) and BF (*p* = 0.034) (Table 3). Total Omega 6 and ARA levels were similar in the 3 formula groups and breastfed BF (Table 3). However, LA levels in formula D-fed infants were similar to formula P-fed (*p* = 0.630) but higher than in formula PDHA-fed (*p* <  0.001) and BF (*p* <  0.001). ALA and EPA levels in formula D-fed infants were significantly higher than in the other groups (all *p* < 0.05).

**Table 3:** RBC membrane FA levels (% of total FA) after 4 months

	Formula D *n* = 23	Formula P *n* = 24	Formula PDHA *n* = 23	BF *n* = 18	*p*-value
Total SFA	33.4 ± 3.1	34.1 ± 3.6	35.2 ± 2.5	34.7 ± 2.9	0.514
Total MUFA	22.4 ± 2.1^a^	21.5 ± 1.5^a, b^	20.2 ± 1.4^b^	21.0 ± 2.1^b^	< 0.001
Total PUFA	43.7 ± 3.6	44.1 ± 3.7	44.3 ± 2.5	43.9 ± 3.5	0.342
Total Omega 6	35.0 ± 2.7	38.3 ± 3.3	35.4 ± 1.8	34.0 ± 2.5	0.465
18:2(n-6) LA	6.2 ± 0.6^a^	6.3 ± 0.6^a^	4.4 ± 0.4^b^	3.8 ± 0.8^c^	< 0.001
20:4(n-6) ARA	20.3 ± 1.9	21.6 ± 2.2	22.6 ± 1.5	22.2 ± 1.7	0.747
22:4(n-6)	5.9 ± 1.1	6.9 ± 1.3	6.2 ± 1.1	5.6 ± 1.5	0.261
22:5(n-6) DPA n-6	0.8 ± 0.2	1.1 ± 0.3	0.9 ± 0.2	0.9 ± 0.3	0.359
18:3(n-3) ALA	0.2 ± 0.0^a^	0.1 ± 0.0^b^	0.1 ± 0.0^b^	0.1 ± 0.1^b^	< 0.001
20:5(n-3) EPA	0.7 ± 0.2^a^	0.3 ± 0.1^b^	0.4 ± 0.1^c^	0.5 ± 0.1^c^	< 0.001

Formula D (*n* = 23) contained a mixture of dairy lipids and plant oils; formula P (*n* = 24) contained only plant oils and formula PDHA (*n* = 23) contained plant oils supplemented with ARA and DHA. BF: breastfed reference group (*n* = 18). PE: phosphatidylethanolamine

Comparison of the 4 groups by ANOVA with 3 fixed factors (Sex, gestational age, group) and post-hoc Tukey’s adjustments

^a^ ≠ ^b^ ≠ ^c^; *p* < 0.05

### Changes in whole blood FA levels between enrollment and 4 months

**Fig. 3:**
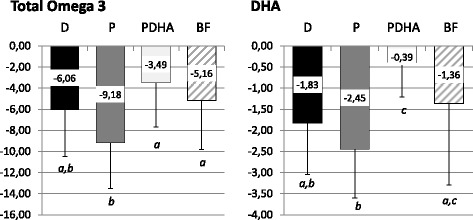
Changes in total Omega 3 and DHA levels in whole blood between 4 months and enrollment expressed as deltas (% of total FA). Formula D (*n* = 23) contained a mixture of dairy lipids and plant oils; formula P (*n* = 24) contained only plant oils and formula PDHA (*n* = 23) contained plant oils supplemented with ARA and DHA. BF: breastfed reference group (*n* = 18). Comparison of the 4 groups by ANOVA with 3 fixed factors (Sex, gestational age, group) and post-hoc Tukey’s adjustments. *a ≠ b ≠ c; p < 0.05*

Changes of total Omega 3 and DHA levels (expressed as deltas) in whole blood between 4 months and enrollment are presented in Fig. 3. Percentage of total Omega 3 significantly decreased between 4 months and enrollment in all groups (Fig. 3). The decrease in percentage of Omega 3 in group D (− 6.1 ± 4.4% TFA) was similar to those in group P (− 9.2 ± 4.3% TFA; *p* = 0.087), PDHA (− 3.5 ± 4.2% TFA; *p* = 0.144) and BF (− 5.2 ± 4.7% TFA; *p* = 0.847) (Fig. 3). However, this decrease was significantly higher in group P than in group PDHA (*p* < 0.001) and BF (*p* = 0.15) (Fig. 3). Proportions of DHA in whole blood also decreased with time in all groups (Fig. 3). The decrease in DHA observed in group D (− 1.8 ± 1.2% TFA) was similar to those in group P (− 2.5 ± 1.2% TFA; *p* = 0.388) and BF (− 1.4 ± 1.9% TFA; *p* = 0.639) but higher than in group PDHA (− 0.4 ± 0.8% TFA; *p* < 0.001) (Fig. 3). n-3 DPA levels were not evaluated in whole blood.

## Discussion

This study demonstrated that 4-month consumption of a formula containing dairy lipids was associated with a higher total Omega 3 level in RBC membrane compared with a formula containing only lipids of plant origin in healthy infants. Total Omega 3 levels reached with a formula containing dairy lipids were similar to those of infants fed with a formula supplemented with DHA and to breastfed infants. Although breast milk remains the best nutritional choice for infants, the results of this study suggest that dairy lipids in infant formulas could stimulate the conversion of Omega 3 FA precursors into long-chain derivatives.

The beneficial effects of dairy lipids on Omega 3 FA conversion have already been described in previous studies in humans [8, 9] or animal models [10–12]. These effects of dairy lipids could be due to several mechanisms resulting from the specific FA composition of dairy fat. Firstly, dairy fat contains high proportions of short and medium chain FA, which are highly oxidized after absorption. Therefore, they may spare ALA from oxidation and consequently favor its partitioning towards desaturation and elongation pathways to form long-chain derivatives. Another mechanism to explain the effects of dairy fat could be a selective gene or post-traductional activation of desaturases and elongases which are the enzymes involved in n-3 conversion. In particular, the dairy lipid-based formula had a higher content of myristic acid than plant oil-based formulas and this FA has been shown to increase delta 6 desaturase activity in vitro [7]. However, these hypothesis would need to be demonstrated.

One limitation of this study was that formulas slightly differed by their LA and ALA contents and by their LA to ALA ratio, which was lower in the formula with dairy lipid (around 6:1) than in formulas with only plant oils (around 10:1). The n-6 to n-3 ratio has been recognized as an important factor driving the conversion of ALA to long-chain derivatives because of the competition between LA and ALA for their desaturation and elongation pathways. This could partly explain the increase in RBC total Omega 3 FA observed with the dairy lipid-based formula. However, previous data in rats showed benefits of dairy lipids versus plant oils on RBC composition and brain DHA accretion for similar ALA contents and LA to ALA ratios in the diets [11].

In this study, the increase in RBC total Omega 3 levels with the dairy lipid-based formula resulted from an increase in all the n-3 FA, with n-3 DPA and DHA representing more than 90% of total Omega 3 in RBC. RBC DHA level after 4 months was higher in infants fed the formula containing dairy lipids than in those fed the formula with only plant oils but remained lower than in infants fed with preformed DHA (formula or breastfed infants). RBC membrane DHA levels in infants fed with a formula supplemented with DHA were significantly higher than those observed in infants fed the similar formula without DHA, suggesting that RBC DHA levels are strongly influenced by the DHA supplementation, as already described in previous studies [16–18].

Results of FA compositions in whole blood showed a decrease in proportions of total Omega 3 and DHA between 4 months and enrollment in all groups, but it was more pronounced in infants fed formula with only vegetable lipids. These results are in concordance with those reported in a previous study showing that the largest postnatal decline in DHA was observed in infants fed nonsupplemented formula [19].

RBC n-3 DPA was the main FA affected by dairy lipids and was increased by 90% compared with the plant oil-based formula while DHA was only increased by 22%. The literature on n-3 DPA is quite limited but available data suggests beneficial health effects of this FA [20]. The preferential accumulation of n-3 DPA in RBC observed in this study could be due to a limited rate of the conversion of n-3 DPA to DHA, mainly in liver, as shown by in vivo studies [20]. On the other hand, n-3 DPA might represent the preferred form of incorporation of n-3 long chain FA in RBC. Indeed, ALA can be elongated and desaturated in a tissue-dependent manner, with a tissue-selective accumulation of its derivatives [21]. For example, in ALA-fed rats, an increase in ALA, EPA and DPA was observed in plasma, liver and heart while an increase in DPA and DHA occurred in the brain [21]. Also, in neonatal baboons who received an oral dose of labeled ALA at 4 weeks of age, the distribution of labeled FA at 6 weeks of age was similar in brain, retina and liver, with DHA being the main form of labeled FA whereas in RBC n-3 DPA predominated [22]. Finally, in rats, RBC n-3 DPA was found to be the most reliable indicator of brain DHA status, more than RBC DHA [11]. Therefore, blood analysis might not reflect the potential elongation seen in other tissue compartments such as brain. As further limitation, through this study we were not able to observe the effects of a formula containing dairy lipids on brain or retina FA composition.

Even if RBC DHA levels represent an interesting parameter to evaluate the effects of dietary interventions, they are not always correlated with functional outcomes. For example, in the DIAMOND study, RBC DHA was positively correlated with visual acuity but not with cognitive parameters in term infants [16, 23]. Consequently, it would be interesting to study the effect of an infant formula containing dairy lipids on functional outcomes in infants to verify previous results obtained in animal models.

## Conclusions

This study showed that a formula containing dairy lipid increased RBC total Omega 3 status in healthy term infants, at levels similar to breastfed babies. Breast milk remains the optimal food for infants. However, when breastfeeding is not possible, modifying lipid quality in formula by adding dairy lipids should be considered as an alternative method to improve infants’ Omega 3 FA status. However, further investigation would be needed to evaluate the effect of a dairy lipid-based formula (supplemented or not with DHA) on functional outcomes such as visual acuity or cognitive development.

## Abbreviations

ALA: Alpha-linolenic acid
ARA: Arachidonic acid
BF: Breastfed
BW: Body weight
DHA: Docosahexaenoic acid
EFA: Essential fatty acid
FA: Fatty acid
formula D: With a mix of plant oils and dairy lipids
formula P: With only plant oils
formula PDHA: With plant oils and supplemented with ARA and DHA
LA: Linoleic acid
PUFA: Polyunsaturated fatty acid
SD: Standard deviation
TFA: Total fatty acids
WHO: World Health Organization

## References

[1] Delplanque B, Gibson R, Koletzko B, Lapillonne A, Strandvik B (2015). Lipid quality in infant nutrition: current knowledge and future opportunities. J Pediatr Gastroenterol Nutr.

[2] Uauy R, Mena P, Wegher B, Nieto S, Salem N (2000). Long chain polyunsaturated fatty acid formation in neonates: effect of gestational age and intrauterine growth. Pediatr Res.

[3] Guesnet P, Alessandri J-M (2011). Docosahexaenoic acid (DHA) and the developing central nervous system (CNS) - implications for dietary recommendations. Biochimie.

[4] Innis SM (2007). Fatty acids and early human development. Early Hum Dev.

[5] González HF, Vicentin D, Giumelli O, Vazzano M, Tavella M (2012). Profile of triacylglycerols and percentage of palmitic acid at the sn-2 in breast milk substitutes. Arch Argent Pediatr.

[6] Innis SM (2011). Dietary triacylglycerol structure and its role in infant nutrition. Adv Nutr.

[7] Jan S, Guillou H, D’Andrea S, Daval S, Bouriel M, Rioux V (2004). Myristic acid increases delta6-desaturase activity in cultured rat hepatocytes. Reprod Nutr Dev.

[8] Dabadie H, Peuchant E, Bernard M, LeRuyet P, Mendy F (2005). Moderate intake of myristic acid in sn-2 position has beneficial lipidic effects and enhances DHA of cholesteryl esters in an interventional study. J Nutr Biochem.

[9] Dabadie H, Motta C, Peuchant E, LeRuyet P, Mendy F (2006). Variations in daily intakes of myristic and alpha-linolenic acids in sn-2 position modify lipid profile and red blood cell membrane fluidity. Br J Nutr.

[10] Delplanque B, Du Q, Agnani G, Le Ruyet P, Martin JC (2013). A dairy fat matrix providing alpha-linolenic acid (ALA) is better than a vegetable fat mixture to increase brain DHA accretion in young rats. Prostaglandins Leukot Essent Fatty Acids.

[11] Du Q, Martin J-C, Agnani G, Pages N, Leruyet P, Carayon P (2012). Dairy fat blends high in α-linolenic acid are superior to n-3 fatty-acid-enriched palm oil blends for increasing DHA levels in the brains of young rats. J Nutr Biochem.

[12] Dinel AL, Rey C, Bonhomme C, Le Ruyet P, Joffre C, Laye S (2016). Dairy fat blend improves brain DHA and neuroplasticity and regulates corticosterone in mice. Prostaglandins Leukot Essent Fatty Acids.

[13] Dinel AL, Rey C, Baudry C, Fressange-Mazda C, Le Ruyet P, Nadjar A (2016). Enriched dairy fat matrix diet prevents early life lipopolysaccharide-induced spatial memory impairment at adulthood. Prostaglandins Leukot Essent Fatty Acids.

[14] Giannì ML, Roggero P, Baudry C, Ligneul A, Morniroli D, Garbarino F (2012). The influence of a formula supplemented with dairy lipids and plant oils on the erythrocyte membrane omega-3 fatty acid profile in healthy full-term infants: a double-blind randomized controlled trial. BMC Pediatr.

[15] Auestad N, Montalto MB, Hall RT, Fitzgerald KM, Wheeler RE, Connor WE (1997). Visual acuity, erythrocyte fatty acid composition, and growth in term infants fed formulas with long chain polyunsaturated fatty acids for one year. Ross pediatric lipid study. Pediatr Res.

[16] Birch EE, Carlson SE, Hoffman DR, Fitzgerald-Gustafson KM, Fu VLN, Drover JR (2010). The DIAMOND (DHA Intake And Measurement Of Neural Development) study: a double-masked, randomized controlled clinical trial of the maturation of infant visual acuity as a function of the dietary level of docosahexaenoic acid. Am J Clin Nutr.

[17] Visentin S, Vicentin D, Magrini G, Santandreu F, Disalvo L, Sala M (2016). Red blood cell membrane fatty acid composition in infants fed formulas with different lipid profiles. Early Hum Dev.

[18] Miller MR, Seifert J, Szabo NJ, Clare-Salzler M, Rewers M, Norris JM (2010). Erythrocyte membrane fatty acid content in infants consuming formulas supplemented with docosahexaenoic acid (DHA) and arachidonic acid (ARA): an observational study. Matern Child Nutr.

[19] Guesnet P, Pugo-Gunsam P, Maurage C, Pinault M, Giraudeau B, Alessandri JM (1999). Blood lipid concentrations of docosahexaenoic and arachidonic acids at birth determine their relative postnatal changes in term infants fed breast milk or formula. Am J Clin Nutr.

[20] Kaur G, Cameron-Smith D, Garg M, Sinclair AJ (2011). Docosapentaenoic acid (22:5n-3): a review of its biological effects. Prog Lipid Res.

[21] Barceló-Coblijn G, Murphy EJ (2009). Alpha-linolenic acid and its conversion to longer chain n-3 fatty acids: benefits for human health and a role in maintaining tissue n-3 fatty acid levels. Prog Lipid Res.

[22] Su HM, Bernardo L, Mirmiran M, Ma XH, Nathanielsz PW, Brenna JT (1999). Dietary 18:3n-3 and 22:6n-3 as sources of 22:6n-3 accretion in neonatal baboon brain and associated organs. Lipids.

[23] Drover JR, Hoffman DR, Castañeda YS, Morale SE, Garfield S, Wheaton DH (2011). Cognitive function in 18-month-old term infants of the DIAMOND study: a randomized, controlled clinical trial with multiple dietary levels of docosahexaenoic acid. Early Hum Dev.

